# Tanshinone IIA enhances the ovarian reserve and attenuates ovarian oxidative stress in aged mice

**DOI:** 10.1002/vms3.811

**Published:** 2022-04-21

**Authors:** Lin Bai, Guozhen He, Chenghai Gao, Hua Yang, Mingxing Li, Yulin Huang, Mahmoud Moussa, Changlong Xu

**Affiliations:** ^1^ School of Basic Medicine Guangxi University of Chinese Medicine Nanning China; ^2^ Nanning Second People's Hospital Nanning China; ^3^ Department of Theriogenology Faculty of Veterinary Medicine Suez Canal University Ismailia Egypt

**Keywords:** hormonal regulation, ovarian reserve, oxidative stress, Tanshinone IIA

## Abstract

**Background:**

Tanshinone IIA (TSA), a major lipophilic component extracted from the roots of *Salvia miltiorrhiza Bunge*, has been widely used in China for its various biological activities. However, its effect on ovarian reserve in aged mice was not studied elsewhere.

**Objectives:**

This study aimed to explore the effect of TSA on the ovarian reserve of aged mice as well as young mice. Forty weeks old mice (N = 40) were considered as aged group compared to 4 weeks old mice (N = 40), and these groups were subdivided into four subgroups (N = 10) to receive different doses of TSA (0, 10, 20, and 40 μg/g/day).

**Methods:**

The effect of TSA was evaluated by counting follicular number by histological examination. Basal serum levels of FSH, LH, E2, and anti‐Mullerian hormone (AMH) were measured by ELISA. Moreover, the expression levels of antioxidant genes (CAT, Nrf2, GPX1), gap junction (Cx37), ERK1/2, and Smad5 family gene were examined at both mRNA (qPCR) and protein levels (western blot).

**Results:**

Follicular number, level of AMH and E2, and the expression of CAT, Nrf2, and GPX1 genes increased significantly (*p* < 0.05) in aged mice administrated with medium (20 μg/g/day) and high (40 μg/g/day) doses of TSA, whereas FSH and LH levels were significantly low compared to low dose (10 μg/g/day) and control (0 μg/g/day) aged subgroups. However, we did not observe any effect of all doses of TSA on young mice.

**Conclusions:**

Administration of TSA with medium and high doses up‐regulates the expression of antioxidative genes, reduces the oxidative injury, increases levels of AMH, and E2 levels that are relatively comparable to those in young mice, and consequently results in a healthy oocyte development.

## Graphical abstract

1. Tanshinone IIA (TSA) enhances the ovarian reserve in aged mice.

2. Administration of medium (20 μg/g/day) and high (40 μg/g/day) doses of TSA attenuates the oxidative

3. TSA improves the serum level of anti‐Mullerian hormone and oestrogen in aged mice.

4. TSA has no any significant effect on the ovarian functions of young mice.

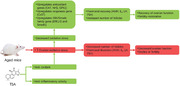

## 1 INTRODUCTION

Female fertility is affected by multiple factors, including genetic, environmental, nutritional, and endocrinological factors. Ovarian function has the major role in maintaining a healthy reproductive function during female lifetime. A common feature in females of most mammalian species is age‐related decline in fertility (Moussa et al., 2015). There are two reasons for this: age‐related follicular and endocrine changes (Malhi et al., 2005). Reproductive aging in cattle is associated with elevated circulating concentrations of gonadotropins, reduced concentrations of steroid hormones, and fewer 4‐ to 5‐mm follicles were recruited into a wave; and, secondly, maternal age‐related mitochondrial dysfunction (Takeo et al., 2012). This dysfunction is due to increased oxidative stress, deletions, or point mutations and variations in the mitochondrial genome (Shamsi et al., 2013; Takeo et al., 2012).

Ovarian reservation (OR) refers to the number and quality of oocytes, and it plays an essential role in achieving pregnancy. Diminished OR is a term used to denote that ovaries have eggs that are lower in number or quality than expected, and is characterized by increased apoptosis rate of granulosa cells (Y. Fan et al., 2019) and poor fertility outcomes (Chang et al., 2018). The condition may result from disease or injury, but most commonly occurs as a result of normal aging. Delayed childbearing is the main challenge in reproductive medicine as increased age has an adverse effect on successful pregnancy, both in natural and assisted reproduction. With the continuous increase of age, the function of ovarian interstitial cells decreases, the proportion of granulosa cell apoptosis increases, and the number and quality of oocytes gradually decrease (Yuan et al., 2016). Unfortunately, there is no specific treatment that can slow ovarian aging and truly prevent diminished ovarian reserve.

Owing to its mild and broad therapeutic efficacy, tanshinone IIA (TSA), a major component extracted from the traditional herbal Chinese medicine *Salvia miltiorrhiza Bunge*, has been widely studied for its various biological activities, including antiangiogenic (Zhou, Sui, et al., 2020), antioxidant (Fu et al., 2007), anti‐inflammatory (Jang et al., 2003), anti‐cancer properties (Chiu & Su, 2010), neuron‐protective (Wang et al., 2018), anti‐atherosclerotic (Tan et al., 2019), antiallergic (Heo & Im, 2019), anticonvulsant (Buenafe et al., 2013), anti‐Alzheimer's disease (Li et al., 2015), reducing organ damage (Ma et al., 2018), attenuating polycystic ovarian syndrome (Jin et al., 2019), suppression of ovarian cancer growth (Zhou, Jiang, et al., 2020), and protection from angina pectoris and myocardial infarction (Lv et al., 2018). Various pre‐clinical and clinical studies have asserted the role of TSA in targeting tumour cells. Its anti‐tumour mechanism may be involved in inhibiting tumour growth, inducing apoptosis, regulating cell cycle, regulating signalling pathways, and reversing the multidrug resistance in various human tumour cells (Kim et al., 2015). It also improves the activity of transcription factors of various antioxidant enzymes and reduces the oxidative stress response (Fu et al., 2007). The combination of TSA and other clinical commonly used drugs could enhance the therapeutic effect of these drugs, which is of great significance to the effective use of TSA in the future (Ansari et al., 2021).

The estimation of diminished ovarian reserve is routinely performed through measurement of hormone levels (Cohen et al., 2015). In this context, serum levels of follicle stimulating hormone (FSH), anti‐Mullerian hormone (AMH), Luteinizing hormone (LH), and Oestradiol (E2) have been suggested as potential markers of fertility (Ramalho de Carvalho et al., 2012; Sharara et al., 1998), among which AMH is suggested as the most sensitive and specific available marker (Kaya et al., 2010; Visser et al., 2012). These hormone levels can show important information about how the ovaries and pituitary gland are working together.

To the best of our knowledge, the effect of TSA on ovarian reserve in aged mice was not studied elsewhere. The aim of the current study was to explore the effect of TSA on the ovarian reserve of aged mice. Follicular number was evaluated by histological examination. Basal serum levels of FSH, LH, E2, and AMH were measured by ELISA. Moreover, the expression levels of antioxidant genes CAT (catalase), nuclear factor erythroid 2‐related factor 2 (Nrf2), glutathione peroxidase (GPX1), gap junction (Cx37), ERK1/2, and Smad5 signal transduction pathway in ovarian tissue were examined at both mRNA (qPCR) and protein level (western blot). The results of this study would offer new possibilities for developing therapeutic strategies in disease state and to enhance female fertility.

## 2 MATERIALS AND METHODS

### 2.1 Experimental animal preparation

The female Kunming mice used in the experiment were purchased from the Experimental Animal Centre. During the experiment, 4 weeks old mice were used as the control group (N = 40), and 40 weeks old mice were considered as the aged group (N = 40). Both groups were subdivided into four subgroups (N = 10) to receive intraperitoneal injection of TSA (Selleck, S2365) at different doses as follow: control (0 μg/gram body weight/day), low dose (10 μg/g/day), medium (20 μg/g/day), and high (40 μg/g/day) doses of TSA. After 2 weeks of daily injection, blood was collected by orbital blood sampling. The peripheral blood of the mouse was centrifuged to separate the serum, and it was frozen for later use. Mice were sacrificed and one ovary was fixed in 4% paraformaldehyde for histological examination, and the other ovary was placed in a cryotube and stored at −80°C for RNA and protein extraction.

### 2.2 Histological examination

The ovarian tissue was fixed with 4% paraformaldehyde for 48 h, then repaired, dehydrated, and embedded, then cut into 5 μm sections, and stained with H&E staining kit (Solarbio, G1121). Follicles were photographed under Nikon ECLIPSE Ni‐E microscope. Five slices of each ovary separated by 20 μm were selected to count the number of follicles.

### 2.3 Quantitative real‐time PCR

Six pools of ovarian tissues for each experimental group were formed. Total RNA was extracted from each pool of by using Trizol® reagent (Invitrogen, 15596018), and a constant amount of RNA (100 ng) was directly reverse‐transcribed into a 20 μl first strand cDNA using a reverse transcription kit (Takara, RR047) following the manufacturer's instructions. Rt‐qPCR was performed in a total volume of 20 μl, containing equally distributed cDNA (100 ng), 10 μM each of the forward and reverse primers, and 10 μl of 2 × SYBR Green Master Mix (SYBR® Premix Ex Taq™ II; Tli RNaseH Plus, Takara, Japan), replenish the remaining volume with nuclease‐free water. All reactions for all genes of interest were performed in triplicate and were run on an ABI 7500 real‐time fluorescent quantitative PCR machine under the following conditions: 95°C for 30 s, followed by 40 cycles at 95°C for 5 s and 58°C for 30 s. Relative quantification of mRNA expression was calculated using the 2^−△△^CT method. The efficiency of qPCR for each gene was calculated using threefold dilution series. The standard curves were created to calculate the slope values, E = 10^(‐1/slope)^, and yielded reaction efficiencies in the range of 91.5%–98.3%. The housekeeping gene GAPDH was used as an internal control to normalize the relative gene. The details of the selected genes and the primer pairs used in the study are provided in Table 1.

**TABLE 1:** Real‐time PCR primers

Name	Primer sequence (5′–3′)	*T*_m_ (°C)	Length	Database
GAPDH	CAGGAGAGTGTTTCCTCGTCC	60.07	222	NM_001289726.1
	TTCCCATTCTCGGCCTTGAC	60.04		
ERK1/2	GTACGGCATGGTCAGCTCAG	60.81	168	NM_011952.2
	CTGAGGATGTCTCGGATGCC	59.97		
Connexin37	CAAGCAGGCGAGAGAGGC	60.51	290	NM_008120.3
	GGTGTGCTGACGAAGAGGAA	59.97		
CAT	AAGATTGCCTTCTCCGGGTG	60.04	163	NM_009804.2
	GACATCAGGTCTCTGCGAGG	59.90		
Smad5	CTTTCTCCTCTGCGCTTCTGG	61.01	157	NM_001164042.1
	TTCTTAGTGCAAGTCCTCGACC	60.03		
Nrf2	AGATGACCATGAGTCGCTTGC	60.74	296	NM_010902.4
	CCAGCGAGGAGATCGATGAG	59.76		
GPX1	CAGTCCACCGTGTATGCCTTC	61.01	233	NM_008160.6
	TCATTCTTGCCATTCTCCTGGT	59.69		

### 2.4 Western blot

Ovarian tissue was lysed in RIPA‐PMSF protein extraction reagent (Invitrogen, 89900), homogenized at low temperature, and centrifuged to obtain a total protein lysate. After the concentration was determined by the bicinchoninic acid assay, the concentration of each histone was adjusted to be consistent. Lysates were diluted with 6 × protein loading buffer (Invitrogen, NP0007) and heated to 100°C for 10 min for thermal denaturation of protein. Samples were then loaded on a 10% gradient polyacrylamide gel (P0012AC, 202 Beyotime, China) and then transferred to a PVDF membrane (ISEQ00011, Millipore). Then, the membrane was blocking in 5% (wt/vol) Difco skim milk in Tris‐buffered saline containing 0.1% (vol/vol) Tween‐20 (TBST) for 2 h. The primary antibody was incubated overnight at 4°C. The detection indicators were as follows: GAPDH (Abcam, ab9485, 1:5000), ERK1/2 (Abcam, ab184699, 1:10000), connexin37 (Abcam, ab181701, 1:10000), CAT (Abcam, ab209211, 1:2000), Smad5 (Abcam, ab40771, 1:1000), Nrf2 (Abcam, ab137550, 1:1000), and GPX1 (Abcam, ab59546, 1:1000). After the primary antibody was incubated, the membrane was washed with PBST, and then the secondary antibody was incubated at 37°C. The secondary antibody used in the experiment was Goat Anti‐Rabbit IgG H&L (HRP, Abcam, ab6721, 1:10000). After 1 h of incubation, the secondary antibody was eluted with PBST, then developed with ECL exposure solution, imaged and photographed in a multi‐function imager. ImageJ 7.0 software was used to read the band grey value, and GAPDH was used as the internal reference genes to normalize the target protein expression.

### 2.5 Measurement of FSH, LH, AMH, and E2 using ELISA

Basal serum level of targeted hormones was tested by Elisa as follows: FSH (Gene beauty, Wuhan, JYM0417Mo), LH (Gene beauty, Wuhan, JYM0341Mo), E2 (Gene beauty, Wuhan, JYM0379Mo), and AMH (Gene beauty, Wuhan, JYM0786Mo). All of the procedures were performed according to the manufacturer's instructions. Optical Density (OD) at 450 nm was measured using MD FilterMX microplate reader. Rregression analysis was used to detemine the hormonal levels of each group against the standard curve of each index.

### 2.6 Statistical analysis

All data in this study were statistically analyzed using SPSS 22.0 software. The experimental test data are expressed in the form of standard mean deviation (mean ± SD), single‐factor ANOVA is used for comparison between multiple groups, and SNK is used for pairwise comparison. *p* ≤ 0.05 indicates that the difference is statistically significant.

## 3 RESULTS

### 3.1 The effect of TSA on the number of follicles in ovarian tissue

**FIGURE 1:**
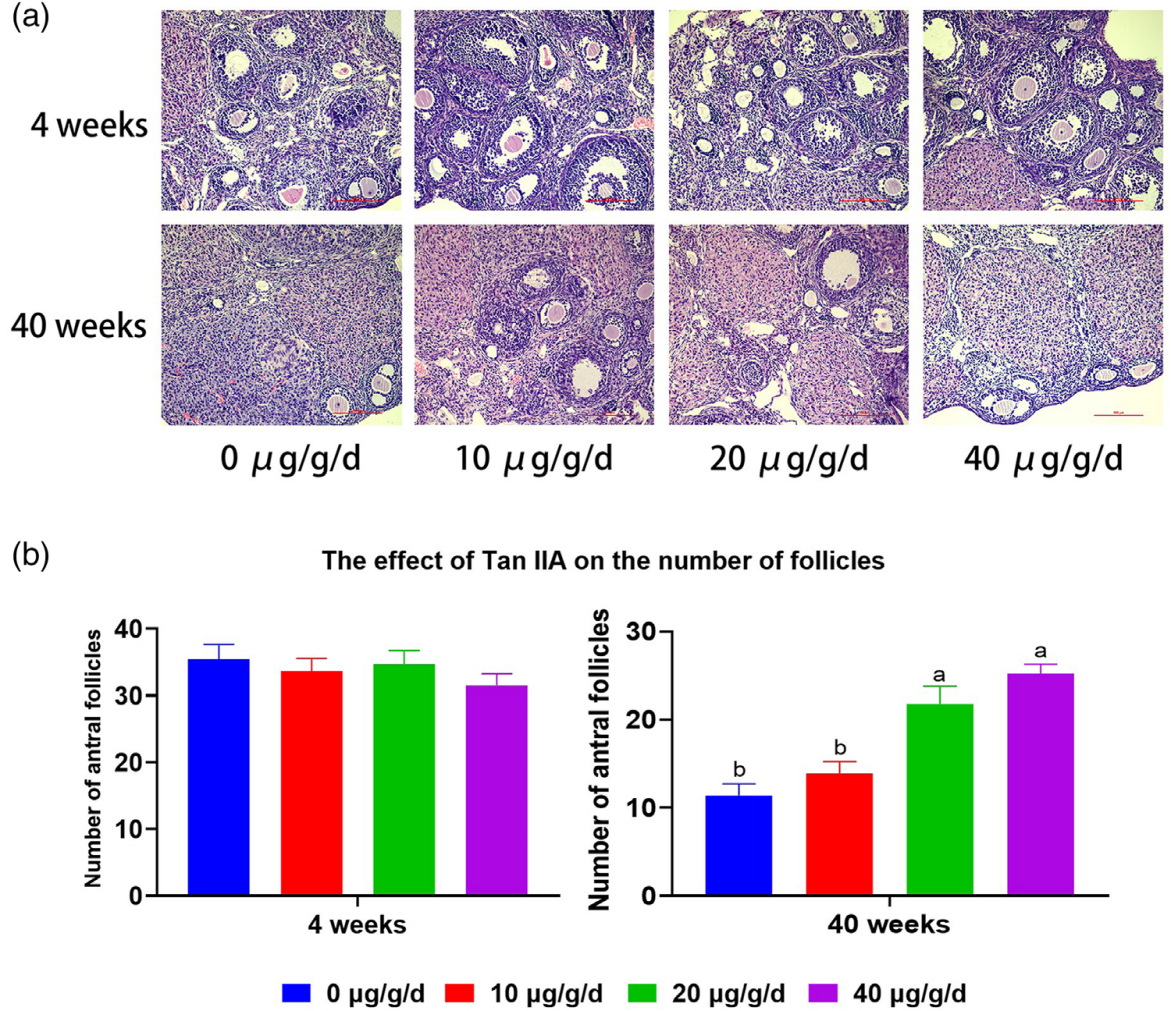
Representative photomicrographs of the ovaries using haematoxylin and eosin (H&E) staining (a), and follicle count statistics (b) of young (4 weeks old) and aged (40 weeks old) mice after administration of different doses of tanshinone IIA (TSA) (0, 10, 20, 40 μg/g/day). Different letters above the bars (a, b, c, and d) indicate significant differences (*p* < 0.05). Scale bar: 100 μm

Following administration of TSA, there was no significant (*p* > 0.05) difference between different doses of TSA in the number of ovarian follicles in the control group (35.4 ± 7.16, 33.6 ± 6.18, 34.7 ± 6.53, 31.5 ± 5.5), indicating that TSA has no effect on the ovarian reserve of young mice. However, administration of high and medium doses of TSA in aged group significantly (*p* < 0.05) increased the ovarian reserve (25.2 ± 3.52, 21.8 ± 6.32) compared to low dose (13.9 ± 4.28) and control aged subgroups (11.4 ± 4.15), respectively (Figure 1).

### 3.2 Hormonal levels in young and aged mice

**FIGURE 2:**
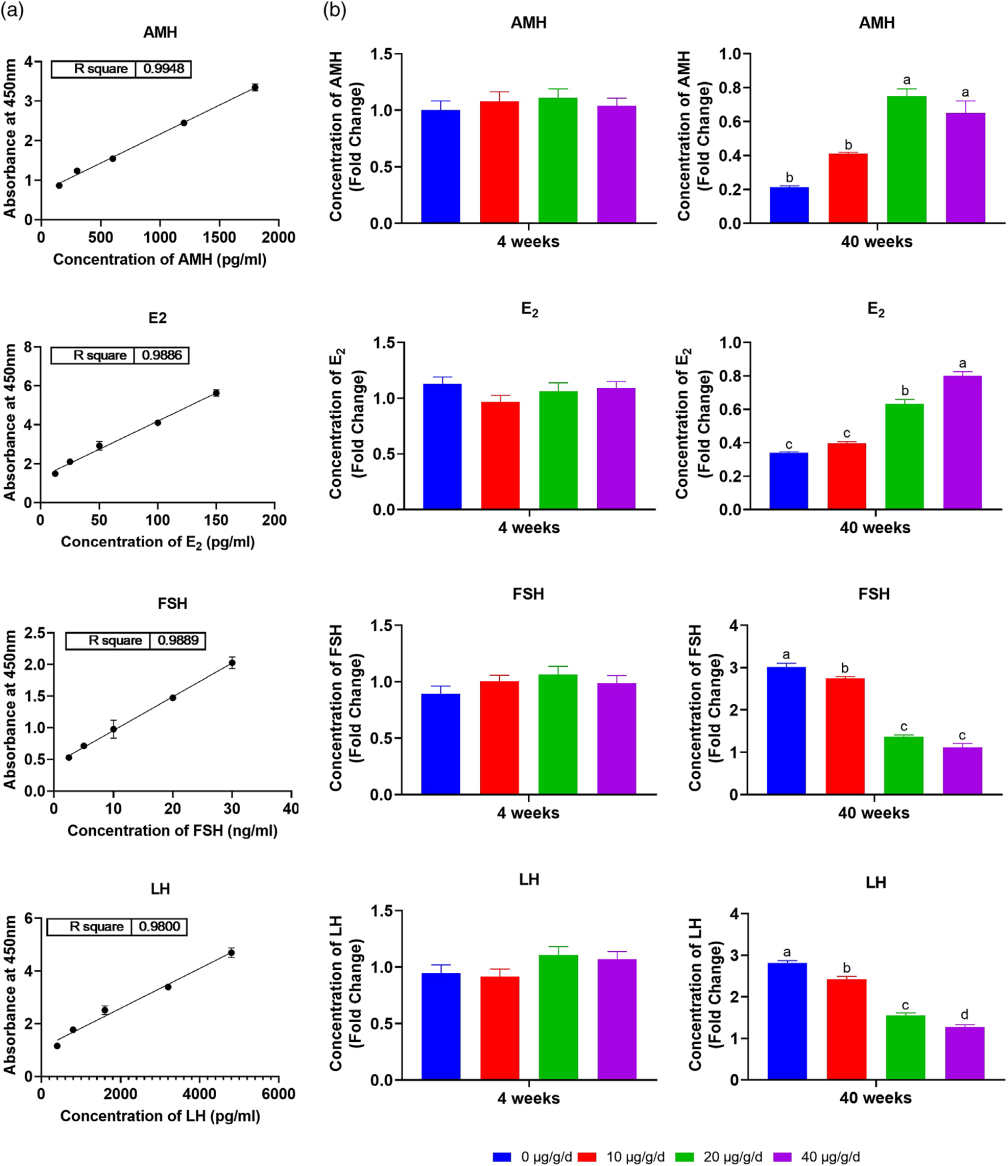
Hormonal level of anti‐Mullerian hormone (AMH), E2, follicle stimulating hormone (FSH), and Luteinizing hormone (LH) in the serum of young (4 weeks old) and aged (40 weeks old) mice after administration of different doses of tanshinone IIA (TSA) (0, 10, 20, 40 μg/g/day). Different letters above the bars (a, b, c, and d) indicate significant differences (*p* < 0.05)

There were no significant (*p* > 0.05) changes in the levels of AMH, E2, FSH, and LH hormones in young mice after administration of different doses of TSA. Although, in aged mice, there were some changes in the levels of E2, FSH, and LH levels between low dose subgroup compared to control subgroup, the difference was not significant (*p* > 0.05). However, the levels of AMH and E2 hormones in the high (0.65 ± 0.22, 0.8 ± 0.08) and medium dose (0.75 ± 0.13, 0.63 ± 0.08) groups were significantly increased (*p* < 0.05) compared to low dose (0.41 ± 0.03, 0.4 ± 0.03) and control (0.21 ± 0.03, 0.34 ± 0.02) aged subgroups. In addition, the levels of LH and FSH were also decreased in high (1.27 ± 0.21, 1.12 ± 0.26) and medium (1.56 ± 0.19, 1.36 ± 0.14) dose groups compared to low (2.42 ± 0.23, 2.75 ± 0.12) and control (2.82 ± 0.17, 3.01 ± 0.28) aged groups, respectively (Figure 2).

### 3.3 The relative abundance of mRNA of ERK1/2, Smad5, Nrf2, and GPX1 in the ovarian tissue of young and aged mice

**FIGURE 3:**
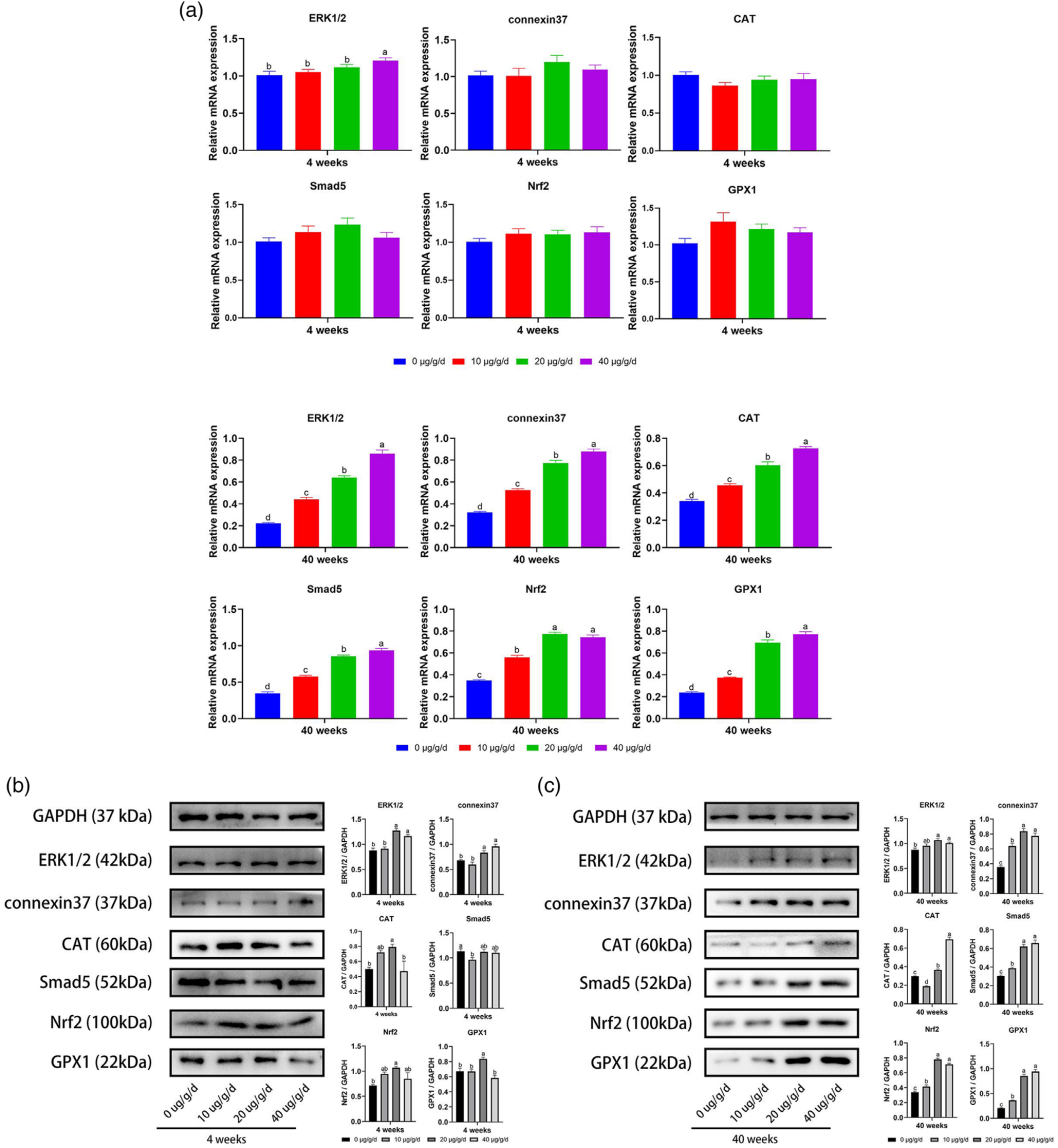
Quantitative RT‐PCR revealed relative mRNA levels (a) and protein expression of ERK1/2, Cx37, CAT, Smad5, Nrf2, and GPX1 in ovarian tissues of young (4 weeks old) (b) and aged (40 weeks old) (c) female mice after administration of different doses of tanshinone IIA (TSA) (0, 10, 20, 40 μg/g/day). The expression of these mRNAs was normalized to the expression level of GAPDH measured in the same RNA preparation. Different letters above the bars (a, b, c, and d) indicate significant differences (*p* < 0.05)

The results showed that there was no significant difference between different doses of TSA on the expression level of ERK1/2, connexin37, CAT, Smad5, Nrf2, and GPX1 in the ovarian tissue of young mice. However, after administration of TSA in aged mice, particularly medium and high doses of TSA, the expression level of ERK1/2, connexin37, CAT, Smad5 and Nrf2 was significantly (*p* < 0.05) up‐regulated compared with the control aged subgroup (Figure 3). The results of protein expression were consistent with that of mRNA. Such results support the major role played by TSA in attenuating the oxidative stress in ovarian tissues of aged mice.

## 4 DISCUSSION

The dried root of *S. miltiorrhiza* is one of the most used herbs in Chinese medicine because of its high value. Growing evidence indicates that natural compounds such as TSA possess remarkable curative effect and minute adverse reactions. TSA is a major lipophilic component of extracts from the root of *S. miltiorrhiza*. It has anti‐inflammatory activity (Jang et al., 2003), antioxidant (Fu et al., 2007), cytotoxic to various cancer cell (Chiu & Su, 2010). Also, it has traditionally been used in the treatment of cardiovascular diseases (Luo et al., 2015) through its antioxidant effect (J. Zhu et al., 2017). More importantly, TSA is a new candidate target to be used as an anti‐tumour drug in clinical practice and contributes to the suppression of ovarian cancer growth (Zhou, Sui, et al., 2020). TSA has also shown promising results pertaining to treatment of polycystic ovarian syndrome in mice (Jin et al., 2019). Studies to identify the molecular and cellular mechanisms will further shed light on the importance of TSA both for the development and function of reproductive tissues as well as disease condition.

**FIGURE 4:**
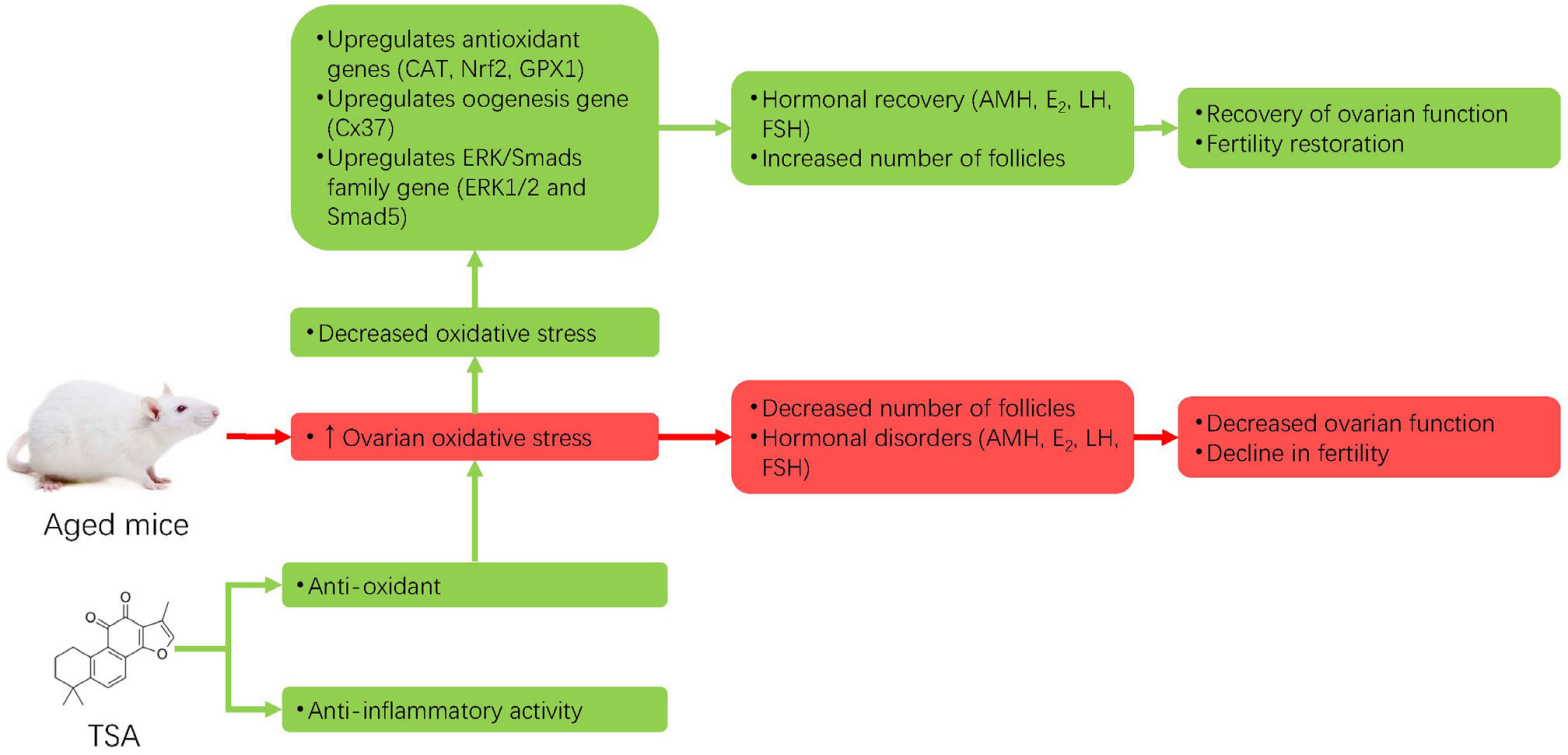
An overall scheme showing the mechanisms by which tanshinone IIA (TSA) enhances the ovarian reserve in aged mice

Previous studies have shown that oxidative stress resulting from aging declines egg quality and ovarian reserve as well, which has a serious impact in the maintenance of female fertility (May‐Panloup et al., 2016; Yang et al., 2021). Thus, alleviating oxidative stress in the ovaries is an important step for delaying ovarian aging. Nrf2 is an important transcription factor for cell self‐antioxidation and plays a major role in controlling the cellular susceptibility to reactive oxygen species (ROS)‐induced cytotoxicity (H. Zhu et al., 2005). Endogenous intracellular antioxidant enzymes provide the primary defence against oxidative stress caused by the accumulation of free radicals. GPX1 and CAT are the most common enzymatic antioxidants and play critical roles in removing the harmful oxygen products produced by superoxide dismutase (Fridovich, 1995). For this reason, equilibration status of oxidative stress is very essential process in reproductive medicine (Akino et al., 2018). High expression of Nrf2 in aged mice after administration of TSA might lead to alleviate oxidative stress in ovarian tissue, and this could provide novel insight to conquer the age‐related fertility decline that is mainly attributed to the accumulation of aberrant oxidative stress. Moreover, it has been investigated that total apoptosis rates were significantly increased in women with diminished ovarian reserve when compared to women with normal ovarian reserve, along with lower AMH level (Y. Fan et al., 2019). Nrf2 is critically involved in the regulation of cell proliferation and apoptosis during the decline of ovarian reserve (Chen et al., 2017). The primary endogenous antioxidant defence enzymes, CAT, and GPX1 are together responsible for protection against the damaging effects of free radicals. In this regard, antioxidants from natural products are recommended because they have a high anti‐oxidative stress capacity and are safe and acceptable compared with synthetic antioxidants (Yang et al., 2021). Since ovarian reserve is an important factor in achieving pregnancy both in natural and assisted reproduction, TSA‐mediated regulation of antioxidants of the ovarian tissue is of importance for protection against ovarian oxidative disorders (Figure 4).

A similar pattern was observed in the aged group is that the levels of gonadotropins level (FSH and LH) increased, whereas the levels of E2 and AMH decreased, indicating a diminished ovarian reserve. In support of our findings, it has been demonstrated that elevated FSH and LH is closely related to the decrease of ovarian function (Takeo et al., 2012). However, in aged mice, administration of medium and high doses of TSA significantly reduced the level of FSH and LH, and increased the levels of E2 and AMH, suggesting that TSA can effectively alleviate the adverse effect of ovarian aging. Furthermore, AMH is a glycoprotein of the TGFβ superfamily, and is suggested as the most specific available marker for ovarian reserve (Kaya et al., 2010; Visser et al., 2012) was found low in aged mice. One of the roles of AMH is to inhibit primordial follicle activation, which slows the rate at which the ovarian reserve is depleted. Elevated AMH level after administration of TSA in aged mice might be one possibility by which TSA promotes the ovarian reserve, but further studies will be necessary to determine the mechanism by which this is achieved.

ERK1/2 and Smad5 family proteins are widely expressed in oocytes and granulosa cells at various developmental stages (Heldin & Moustakas, 2012; Sakaki‐Yumoto et al., 2013) and are very essential for ovarian functions. ERK1/2 are necessary for LH‐induced oocyte resumption of meiosis, ovulation, and luteinization (H.‐Y. Fan et al., 2009). Moreover, defect in Smad5 family proteins expression is known to have adverse effects on ovarian organogenesis and folliculogenesis (Kaivo‐oja et al., 2006). The expression level of these genes was significantly decreased in aged mice; however, administration of TSA particularly with medium and high doses improved the expression levels of ERK1/2 and Smad5 family at both mRNA and protein levels, suggesting that TSA maintains the ovarian functions through different ways.

Connexin 37 is critical to ovarian function as it participates in sustaining proper growth and maturation of the oocyte and plays an important role in folliculogenesis (Teilmann, 2005). A targeted mutation of Cx37 induces female infertility (Gittens & Kidder, 2005). Similarly, in sheep ovary, it plays a role in follicular development and ovulation (Borowczyk et al., 2006). In this regard, we infer that aging has an adverse effect on the expression of Cx37 which may explain, at least in part, the low fertility of aged female mammals. The high expression of Cx37 at both mRNA and protein levels after TSA administration could inspire relevant researches for its potential medicinal value. Taken together, these findings argue for a supportive role of TSA during ovarian ageing in female mammals that may be of value clinically. The reasons for the positive effect of TSA on ovarian reserve of aged but not in young mice remain unclear and require further investigation. However, the higher accumulation of ROS of the aged mice could render them in an oxidative stress that is diminished by the TSA administration.

In conclusion, despite the important development in ART and the improvement in our understanding of the maturation events, accurate selection of good‐quality oocytes of aged ovary is particularly challenging. Administration of TSA with medium and high doses up‐regulates the expression of antioxidative genes, reduces the oxidative injury, and consequently results in a healthy oocyte development. Another way by which TSA affects the ovarian reserve in aged mice is through an increase in the level of AMH and E2 the most specific available markers for ovarian reserve. In‐depth research on TSA has attracted a great deal of attention worldwide, which may expand the effective clinical uses of the underlined compound and its different dosage forms. Obviously, controlled studies with the use of TSA must be conducted to validate these promising results.

## CONFLICT OF INTEREST

The authors declare no conflict of interest.

## ETHICS STATEMENT

All animal procedures used in this study were carried out in accordance with the Guide for Care and Use of Laboratory Animals (8th edition, released by the National Research Council, USA) and were approved by the Institutional Animal Care and Use Committee (IACUC) of Guangxi University of Chinese Medicine.

## AUTHOR CONTRIBUTIONS

Lin Bai, Hua Yang, Mingxing Li, Yulin Huang performed the experiments and drafted the manuscript, Guozhen He and Chenghai Gao analyzed the data, Mahmoud Moussa wrote the manuscript, and Changlong Xu designed the study.

### PEER REVIEW

The peer review history for this article is available at https://publons.com/publon/10.1002/vms3.811.

## Data Availability

The data that support the findings of this study are available from the corresponding author upon reasonable request.
